# LINC-complex mediated positioning of the vegetative nucleus is involved in calcium and ROS signaling in Arabidopsis pollen tubes

**DOI:** 10.1080/19491034.2020.1783783

**Published:** 2020-07-07

**Authors:** Morgan Moser, Andrew Kirkpatrick, Norman Reid Groves, Iris Meier

**Affiliations:** a Department of Molecular Genetics, The Ohio State University, Columbus, OH, USA; b Center for Applied Plant Sciences, The Ohio State University, Columbus, OH, USA; c Center for RNA Biology, The Ohio State University, Columbus, OH, USA

**Keywords:** Male fertility, pollen tube termination, nuclear envelope, LINC complex, nuclear calcium, reactive oxygen species

## Abstract

Nuclear movement and positioning play a role in developmental processes throughout life. Nuclear movement and positioning are mediated primarily by linker of nucleoskeleton and cytoskeleton (LINC) complexes. LINC complexes are comprised of the inner nuclear membrane SUN proteins and the outer nuclear membrane (ONM) KASH proteins. In Arabidopsis pollen tubes, the vegetative nucleus (VN) maintains a fixed distance from the pollen tube tip during growth, and the VN precedes the sperm cells (SCs). In pollen tubes of *wit12* and *wifi*, mutants deficient in the ONM component of a plant LINC complex, the SCs precede the VN during pollen tube growth and the fixed VN distance from the tip is lost. Subsequently, pollen tubes frequently fail to burst upon reception. In this study, we sought to determine if the pollen tube reception defect observed in *wit12* and *wifi* is due to decreased sensitivity to reactive oxygen species (ROS). Here, we show that *wit12* and *wifi* are hyposensitive to exogenous H_2_O_2_, and that this hyposensitivity is correlated with decreased proximity of the VN to the pollen tube tip. Additionally, we report the first instance of nuclear Ca^2+^ peaks in growing pollen tubes, which are disrupted in the *wit12* mutant. In the *wit12* mutant, nuclear Ca^2+^ peaks are reduced in response to exogenous ROS, but these peaks are not correlated with pollen tube burst. This study finds that VN proximity to the pollen tube tip is required for both response to exogenous ROS, as well as internal nuclear Ca^2+^ fluctuations.

## Introduction

Nuclear movement and positioning are vital for cellular processes and development in opisthokonts, for example for p-cell and hypodermal cell development in *C. elegans* and eye disc development in *D. melanogaster* [reviewed in 1, 2]. In vertebrates, nuclear movement and positioning are required for myofiber development and formation of neuromuscular junctions [1]. Nuclear movement in opisthokonts is primarily mediated by two groups of proteins: the outer nuclear membrane (ONM) Klarsicht/ANC-1/Syne Homology (KASH) proteins, and the inner nuclear membrane (INM) Sad1/Unc-84 (SUN) proteins [2]. SUN and KASH proteins bind in the nuclear envelope (NE) lumen and form the core of Linker of Nucleoskeleton and Cytoskeleton (LINC) complexes [1]. KASH proteins, which have variable cytoplasmic domains, interact directly or indirectly with a variety of cytoskeletal elements, while SUN proteins primarily interact with Lamin A/C [2].

While plants have homologs of SUN proteins, they lack homologs of opisthokont KASH proteins. Approximately 40 amino acids of the C-terminus of animal KASH proteins extend into the NE lumen and terminate in a consensus ‘PPPX’ motif [2]. Plant KASH proteins were first identified in Arabidopsis, through identification of a transmembrane domain, a shorter luminal tail domain (9–15 aa), and a similar characteristic C-terminal tail, terminating in a consensus ‘XVPT’ motif [3,4]. Homologs of Arabidopsis SUN and KASH proteins have subsequently been found in a variety of land plant species [5] and functionally investigated in *Z. mays* and *M. truncatula* [4,6,7,8]. The first identified plant KASH proteins, WPP-Interacting Proteins (WIPs) 1, 2 and 3, along with their ONM interaction partners the WPP-Interacting Tail Anchored Proteins 1 and 2 (WITs), form a LINC complex with Arabidopsis SUN1 and SUN2 [3]. This complex binds a motor protein, Myosin XI–i, and is involved in moving nuclei in root and leaf cells [9]. In pollen, the WIT-WIP-SUN complex is involved in male fertility. The WIP triple mutant *wip123* and WIT double mutant *wit12* have a random seed-loss phenotype [4]. Loss of either the WIP or WIT protein family, as well as loss of both, results in about 50% reduction in seed set [10]. While pollen tube growth is not significantly altered in these mutants, the distance of the pollen vegetative nucleus (VN) from the growing pollen tube tip is increased [10]. The resulting mutant pollen tubes have defects in pollen tube termination, with pollen tube overgrowth at the site of ovules frequently observed [10].

During pollen germination and subsequent pollen tube growth in many angiosperm species, the two sperm cells (SCs) are physically connected to the VN, and collectively named the male germ unit (MGU) [11]. As the pollen tube grows, the MGU maintains a fixed distance from the advancing pollen tube tip, with the VN leading the SCs [12]. The fact that the VN and SCs migrate as a unit has been proposed to be important for efficient movement of the SCs to the ovule [13,15]. A mutant deficient in two helix-loop-helix transcription factors, *drop1 drop2*, transports only the VN and is phenotypically near normal, including pollen tube rupture after entering the ovule [16]. This suggests that reception and termination signaling can proceed in the absence of the SCs.

In contrast, *wip* and *wit* mutant pollen tubes that transport SCs, but lead to a partial loss of the VN at the pollen tube tip, have defects in pollen tube termination [10]. Upon mutant pollen germination, the SCs emerge first, followed by the VN, a reversal in MGU order compared to wildtype [10]. *Wip* and *wit* mutant pollen tubes grow at normal rates, but the distance of the VN to the pollen tube tip increases over time [10]. Frequently, the VN is not observed at the pollen tube tip, and the corresponding pollen tubes either stall at the entrance to an ovule or continue to grow past the synergids but remain intact [10,17]. SUN1 and SUN2 mutant combinations of varying severity are defective in male gametophyte development [18] or show reduced seed set [19]. A combination of knockdown, knockout, and a dominant-negative approach presented the same MGU order defects seen in *wip* and *wit* mutants, indicating a role for SUNs in this process [19]. How the requirement for the WIT-WIP-SUN complex, and, by extension, the tip-located VN, relates to the known signaling steps of pollen tube reception and termination is not known.

Pollen tube reception at the ovule involves intricate interactions between the pollen tube and the synergid cells, leading to pollen tube growth arrest and burst, and the release of the SCs [20,21]. Several signaling components of this step on the female side have been identified (for review see [22]). Loss of function mutants of the synergid cell surface receptor kinase FERONIA lead to failed WT pollen tube burst upon arrival at a *fer* mutant ovule, and subsequent pollen tube overgrowth [23]. FERONIA is required for an increase in reactive oxygen species (ROS) production by the synergid cells upon pollen tube arrival and a synergid calcium (Ca^2+^) fluctuation that precedes a corresponding Ca^2+^ increase in the pollen tube tip prior to burst [24,25]. *In vitro*-induction of ROS leads to pollen tube growth arrest and rupture in a Ca^2+^-dependent manner, suggesting pollen tube-synergid crosstalk involves a synergid-triggered Ca^2+^-ROS signal that leads to pollen tube termination and SC release [26].

How signals from the synergids are perceived by the pollen tube remains an open question. Two FERONIA-related proteins, ANXUR1 and ANXUR2, are specifically expressed in pollen, and the double mutant shows premature growth arrest and burst, but whether they are involved in signal reception by the pollen tube is not known [27]. A second pair of pollen tube receptors (BUPS1 and BUPS2) has a similar function, protecting the pollen tube from premature burst. BUPS1 and BUPS2 bind both pollen-expressed and female-expressed Rapid Alkalinization Factor (RALF) peptide ligands, and interaction with female-expressed RALF34 releases the protection from burst by BUPS1 and BUPS2 [28]. Thus, an interplay between ovule-derived and pollen-derived peptide ligands might enable the pollen tube to respond to the synergid environment created by FERONIA and related pathways [28]. The pollen tube response pathway downstream of plasma membrane-associated signaling is less well understood.

Here, we hypothesized that the WIT-WIP-SUN LINC complex is required for ROS-induced pollen tube rupture. We report a decrease in Ca^2+^-dependent ROS-induced pollen tube rupture of *WIP* and *WIT* locus mutants grown semi-*in vivo*. The hyposensitivity to ROS correlates with an increased distance of the VN from the pollen tube tip. In addition, we provide a first report of nuclear Ca^2+^ fluctuations in a gametophytic nucleus and show that the patterns during growth and ROS-mediated pollen tube burst are altered in the mutants, dependent on VN positioning.

## Materials and methods

### Plant materials

*Arabidopsis thaliana* (Columbia-0 ecotype) was germinated on Murashige and Skoog medium plates (Caisson Laboratories) containing 1% sucrose under constant light. Plants at the two-leaf stage were transplanted to soil and grown at an average temperature of 22–23°C under a 16-hour light/8-hour dark regime. The *wit1-1* (GABI-Kat 470E06) *wit2-1* (SALK_127765) (*wit12*) double null mutant was reported by [29] and the quintuple null mutant *wip1-1* (SAIL_390_A08) *wip2-1* (SALK_052226) *wip3-1* (GABI-Kat 459H07) *wit1-1 wit2-1* (‘*wifi’*) mutant was reported by [3]. Heterozygous *male sterility-1* (*ms-1*) was obtained from the Arabidopsis Biological Resource Center (https://abrc.osu.edu/).

### Cloning

All primers used in cloning and construct generation are outlined in Supplemental Table 1. The pollen-specific promoter LAT52 was cloned from the *LAT52_pro_::GFP* construct in the binary plasmid pMDC107 as previously reported [10]. Restriction sites for enzymes *Sac*I and *Spe*I were added to the 5ʹ and 3ʹ ends, respectively. The amplified fragment was digested with the appropriate restriction enzymes. The LAT52 promoter fragment was isolated, purified with the QIAquik PCR Purification kit (Qiagen) and subsequently ligated into a pH2GW7 vector [30].

The Yellow Cameleon 3.6 (YC3.6) calcium (Ca^2+^) sensor N-terminally tagged with the SV40 nuclear localization signal (NLS) was cloned from a *UBQ10_pro_::NLS-YC3.6* [31]. The R-GECO1 Ca^2+^ sensor was amplified from *CMV_pro_::R-GECO1* [32]. NLS-YC3.6 and R-GECO1 were cloned into pENTR/D-TOPO vectors (Life Technologies) and then moved to *LAT52_pro_::pH2GW7* via the Gateway® LR reaction (Life Technologies).

### Generation of transgenic plants

Binary vectors containing Ca^2+^ sensors were transformed into either *Agrobacterium tumefaciens* strains ABI or GV3101 by triparental mating [33]. Arabidopsis plants were transformed using the *Agrobacterium*-mediated floral dip method [34]. The Col-0 ecotype (WT), *wit12* and *wifi* backgrounds were used for the transformation. Transgenic plants were isolated on MS plates supplemented with using 30 µg/mL hygromycin, and positive transformants were confirmed by confocal microscopy. One homozygous T2 transgenic line for each background was used for all assays described here.

### *Semi* in-vivo *pollen germination and ROS-induced pollen tube rupture*

Pollen germination media (PGM) containing 5 mM KCl, 5 mM CaCl_2_, 1 mM Ca(NO_3_)_2_, 1 mM MgSO_4_, 10% sucrose, 0.01% boric acid, pH 7.5 [35] was heated with 0.4% (w/v) agarose, pipetted onto a glass slide, and allowed to solidify before use. Anthers at time of anthesis were used to pollinate stigmas of *ms-1* flowers at developmental stage 14 [10]. Two hours after pollination, stigmas were excised and placed horizontally onto the solid PGM agar pad. The stigmas were incubated for an additional 3 or 5 hours in a humidity chamber [35]. Elongating pollen tubes were imaged using a 4 x objective with 5 times digital zoom. At time of imaging, pollen tubes were treated with 10 µL of liquid PGM (control) or with 10 µL of liquid PGM containing 6 mM H_2_O_2_. Immediately following treatment, single plane images of pollen tubes were acquired every 10 seconds for 10 minutes with confocal microscopy (Eclipse C90i; Nikon). In addition, after this 10-minute time course, images were acquired at different focal planes to ensure that all pollen tubes could be identified and counted. To quantify the percentage of rupture events, the total number of intact and germinated pollen tubes were tabulated prior to and 10 minutes after treatment. The number of pollen tube discharge events was then counted. For the Ca^2+^ depletion experiments, pollen was germinated on low-Ca^2+^ PGM medium (5 mM KCl, 100 µM CaCl_2_, 1 mM MgSO_4_, 10% sucrose, 0.01% boric acid, pH 7.5). Pollen tubes were then treated with 10 µL of low-Ca^2+^ PGM containing 100 µM GdCl_3_ and after a 10-minute incubation 10 µL of low-Ca^2+^ PGM with 12 mM H_2_O_2_ was added.

### Time to rupture quantification

**Figure 1.**
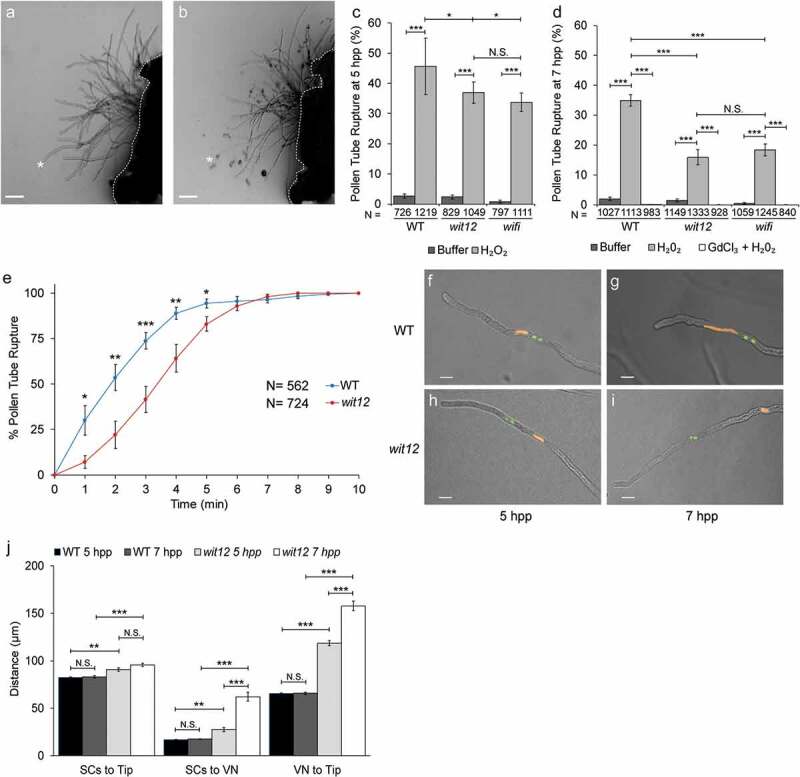
Ca^2+^-dependent ROS-induced pollen tube burst correlates with nuclear position.

The time to rupture was quantified using data collected in Figure 1d. For each experiment, the 10-minute videos were split into 1-minute intervals and the number of pollen tubes that burst during each interval was recorded. That number was then divided by the total number of pollen tubes that ruptured over the course of the 10-minute video and represented as percent. The average percent rupture for each time point was plotted.

(a–b), Semi*-in vivo* germinated WT pollen expressing *Lat52_pro_::R-GECO1* Ca^2+^ sensor. Size bars = 50 µm. (a), Pollen tubes prior to incubation with the ROS generating compound H_2_O_2_. (b), Pollen tubes following incubation with H_2_O_2_. Several tubes have ruptured as indicated by cytoplasm outside the pollen tube. The asterisks in a and b mark the same pollen tube before and after rupture. The white dotted line marks the base of the stigma. (c**–**d), ROS induced pollen tube rupture in semi-*in vivo* germinated pollen at 5 hours (c) and 7 hours (d) post pollination (hpp). Values represent the average number of ruptured pollen tubes observed when treated with buffer, H_2_O_2_, or H_2_O_2_ and a Ca^2+^ channel inhibitor, gadolinium(III) chloride (GdCl_3_ + H_2_O_2_). Bars are standard error. N equals total number of pollen tubes. * P ≤ 0.05; **P ≤ 0.01; *** P ≤ 0.001 for Student’s t-test; N.S. indicates no significance by Student’s t-test. (e), The time to ROS induced pollen tube rupture in semi-*in vivo* germinated WT and *wit12* pollen at 7 hpp. Bars are standard error. N equals total number of pollen tubes. * P ≤ 0.05; **P ≤ 0.01; *** P ≤ 0.001 for Student’s t-test. (f**–**i), The localization of the VN (orange) and SCs (green) was examined in elongating pollen tubes at 5 and 7 hpp. The VN and SCs were visualized using *UBQ10_pro_::NLS-mCherry* and *MGH3_pro_::GFP* respectively. The position of the VN tip in WT remains close to the pollen tube tip at 5 (f) and 7 hpp (g). The VN in *wit12* pollen increases in distance from the pollen tube tip over time. The nucleus is closer at 5 hpp (h) than at 7 hpp (i). Scale bars = 10 µm. (j), The position of the SCs and VN relative to the tip and distance between the SCs and VN was quantified for WT and *wit12* pollen tubes at both time points. N = 150 pollen tubes. Bars are standard error. **P ≤ 0.01; *** P ≤ 0.001 for Student’s t-test.

### Measurement of positions of male germ unit components within the pollen tube

WT and *wit12* lines expressing both the VN marker *UBQ10_pro_::NLS-mCherry* and SC marker *MGH3_pro_::GFP* were germinated using the semi-*in vivo* method on PGM agar pads, as described above. Elongated pollen tubes were imaged using a 40X water immersion objective. Images of pollen at 5 and 7 hpp were acquired at several different focal planes to ensure the MGU and pollen tube tip were in focus. Distances from the relative center of the sperm cells (SCs) and vegetative nucleus (VN) to the tip of the pollen tube were measured using the NIS Elements analysis software (Nikon).

### Ca^2+^ sensor imaging

Pollen expressing either the R-GECO1 Ca^2+^ sensor or the NLS-YC3.6 nuclear Ca^2+^ sensor was germinated as previously described [36]. A square was drawn onto a glass slide with a grease pencil (Staples). An isolated, pollinated stigma was placed in the center of the square. The square was filled with liquid PGM until the media began to mound. The slide was placed in a high humidity chamber for 5 hours to allow pollen tube elongation [36,37]. Individual elongating pollen tubes were imaged under a coverslip with a 40 x water immersion objective with 2 x digital zoom. NLS-YC3.6 sensor was excited with 457 nm, and fluorescence emission was detected between 465 and 505 nm (CFP) and between 530 nm and 570 (cpVenus). R-GECO1 was excited with 561 nm, and its emission was detected between 620 and 650 nm. Time-lapse movies of the pollen tube tip and VN were generated by acquiring an image every 3 seconds for a total of 10 minutes. To quantify the Ca^2+^ fluctuations, a region of interest (ROI) was defined using the binary editor function of the NIS Elements analysis software. For cytoplasmic Ca^2+^, an ROI was drawn proximal to the pollen tube tip. For nuclear Ca^2+^, an ROI was drawn around the VN. The mean fluorescence intensity was obtained for the defined ROI for every image captured in the time-lapse dataset and graphed as a function of time. For NLS-YC3.6, the YFP/CFP ratio for each time point was calculated by dividing the mean fluorescence intensity of YFP by the mean fluorescence intensity of CFP.

### Peak number and standard deviation measurement

Changes in Ca^2+^ sensor fluorescence signals during pollen tube growth were quantified based on the number of peaks and the standard deviation of the RFP signal for R-GECO1 and the YFP/CFP ratio for NLS-YC3.6. Cytoplasmic peaks were determined based on an RFP fluorescence intensity increase of 150 A.U. or higher from the baseline, while nuclear Ca^2+^ peaks were determined based on a YFP/CFP ratio increase of 0.25 A.U. or higher. The average fluorescence intensity or YFP/CFP ratio and the standard deviation for each 200-frame time-lapse movie was calculated. All 20 standard deviations were then averaged to determine the mean standard deviation for R-GECO1 in WT, R-GECO1 in *wit12*, NLS-YC3.6 in WT, and NLS-YC3.6 in *wit12*. Nuclear Ca^2+^ fluctuations after addition of H_2_O_2_ were analyzed using a similar peak determination as described above. However, a nuclear Ca^2+^ peak was determined based on a YFP/CFP ratio increase of 0.50 A.U. or higher.

### Circularity index measurement

WT and *wit12* pollen tubes expressing *Lat52_pro_*::NLS-YC3.6 were grown using the semi-*in vivo* method and imaged at 7 hpp with a confocal microscope (Eclipse C90i, Nikon). Images were taken using a 40 x water immersion objective and acquired using NIS-Elements AR version 3.2. At least 150 pollen vegetative nuclei were visualized for each line by creating z-stack sections (3 μm) to capture the entire nucleus. A maximum intensity projection of each nucleus was generated, and ImageJ was used to calculate the circularity index of each nucleus.

## Results

### Ca^2+^-dependent ROS-induced pollen tube burst correlates with nuclear position

This study uses two previously described Arabidopsis mutant lines ‘*wit12’* (T-DNA insertions in *WIT1* and *WIT2*) and ‘*wifi’* (T-DNA insertions in *WIP1, WIP2, WIP3, WIT1*, and *WIT2*) that have defects in MGU trafficking and pollen tube rupture [10]. Pollen tubes were tested for their sensitivity to external application of hydrogen peroxide (H_2_O_2_) by performing semi*-in vivo* pollen tube rupture assays at 5 hours post pollination (hpp) and at 7 hpp, as described previously [26]. Pollen tube rupture events were defined by the appearance of cytoplasmic material at the end of the pollen tube (Figure 1a,b), as described before [26]. At 5 hpp, approximately 45% of Columbia-0 wild type (WT) pollen tubes treated with H_2_O_2_ burst, corroborating prior studies [26]. In contrast, a reduced number of rupture events were observed in *wit12* and *wifi* pollen, with a burst frequency of 37% and 34%, respectively (Figure 1c).

When the experiment was extended by another 2 hours (7 hpp), efficiency of burst dropped in WT pollen tubes to 35% but dropped substantially more in *wit12* (16%) and *wifi* (18%). At both 5 hpp and 7 hpp, the two mutants behaved similarly (Figure 1c,d). This suggests that semi*-in vivo* pollen tube burst in *wit12* and *wifi* is hyposensitive to external ROS, and that this effect increases over time. When pollen tubes were grown on low calcium (Ca^2+^) media and treated with the Ca^2+^ channel inhibitor gadolinium(III) chloride prior to the application of H_2_O_2_, all three lines behaved similarly with few to no rupture events observed (Figure 1d). In addition, we analyzed time to response of pollen tubes rupture within 10 minutes after addition of H_2_O_2_ (Figure 1e). The data show that there was a delay in response to H_2_O_2_ application in the *wit12* pollen tube population compared to WT.

Together, these data show that *wit12* and *wifi* are similarly hyposensitive to ROS-induced pollen tube rupture, that this hyposensitivity increases over time post-pollination, and that while pollen tubes burst less frequently in *wit12* and *wifi*, the burst is still dependent on Ca^2+^.

Because mutations in *WIP* and *WIT* affect the mobility of the VN during pollen tube growth, we tested if there is a connection between nuclear position and the ROS response. Because *wit12* and *wifi* pollen tubes showed a comparable response to ROS, as well as comparable male fertility defects [10], we proceeded with the *wit12* mutant only. WT and *wit12* pollen tubes concurrently expressing the VN (*UBQ10pro::NLS-mCherry*) and SC (*MGH3pro::GFP*) fluorescent markers were germinated using the semi-*in vivo* method and the nuclear position was examined. In WT, the VN remained at a constant distance of approximately 65 µm from the pollen tube tip, regardless of when the pollen tubes were examined (Figure 1f,g). By contrast, at 5 hpp the VN of *wit12* pollen tubes was positioned, on average, 118 µm from the pollen tube tip (Figure 1h,j). This distance was further increased to an average of 157 µm at 7 hpp (Figure 1i,j). The separation between VN and SCs in *wit12* also increased between 5 hpp and 7 hpp (Figure 1j). Taken together, these data indicate a correlation between VN-tip distance and responsiveness to exogenous ROS in the pollen-tube rupture mechanism.

### *Cytoplasmic Ca^2+^ fluctuations are not disrupted in* wit12 *pollen tubes.*

**Figure 2.**
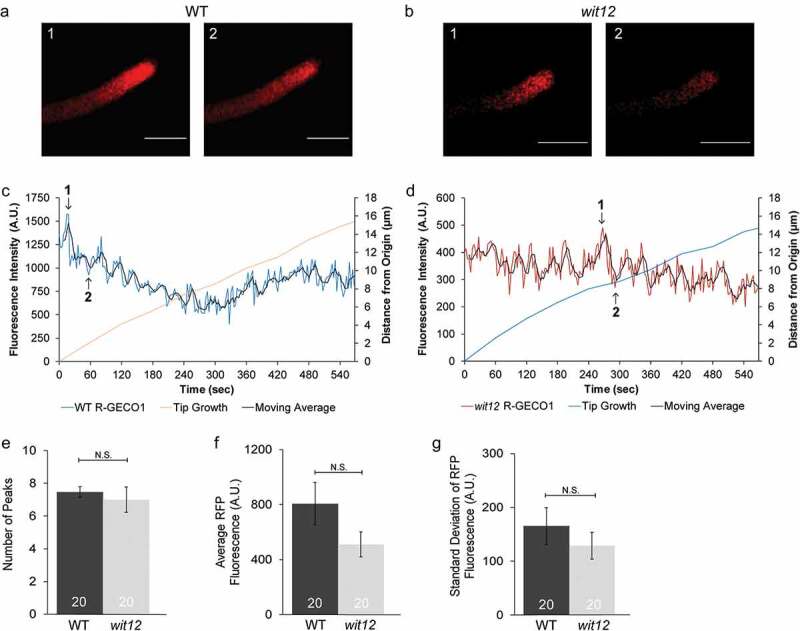
Cytoplasmic Ca^2+^ fluctuations are not disrupted in *wit12* pollen tubes.

Given inhibition of Ca^2+^ channels prevents pollen tube burst in both WT and *wit12*, we next sought to determine if Ca^2+^ signaling was disrupted in *wit12*. Transgenic lines expressing the cytoplasmic Ca^2+^ sensor R-GECO1 under the pollen-specific promoter Late Anther Tomato 52 (LAT52) [38] were used in time-lapse imaging of WT and *wit12* pollen tubes at 7 hpp (Figure 2a,b). Transgenic pollen was germinated using the semi-*in vivo* method. The fluorescence was measured at the tips of growing WT and *wit12* pollen tubes using a region of interest (ROI)-based analysis method in the NIS Elements analysis software (see Materials and Methods). Qualitatively, both WT and *wit12* pollen tubes exhibited a fluorescence pattern (Figure 2c,d) consistent with what has been previously reported for growing Arabidopsis pollen tubes [39]. To determine differences in Ca^2+^ oscillations between WT and *wit12*, the graphs were quantified in two ways. First, the number of peaks over the 10-minute imaging period was counted (Figure 2e). A peak was recorded if an RFP fluorescence intensity both increased and decreased at least 150 A.U. compared to the baseline (see Materials and Methods). WT and *wit12* exhibited similar peak numbers, with an average of 7 peaks within 10 minutes of imaging (Figure 2e). Second, we calculated the average fluorescence intensity and standard deviation of each graph (Figure 2f,g). Here, standard deviation was used as an indicator of how much the fluorescence intensity fluctuated over time compared to the baseline (average fluorescence intensity), with a high standard deviation indicating more fluctuation and a low standard deviation indicating little, if any, fluctuation. Similar standard deviations between WT and *wit12* were observed and correspond with peak frequency (Figure 2g). We observed no significant differences in average fluorescence intensity between pollen tubes (Figure 2f), indicating that expression levels did not significantly influence our results. These data suggest that the cytoplasmic Ca^2+^ oscillations at the pollen tube tip of *wit12* are not significantly altered during elongation.

(a**–**g), The cytoplasmic Ca^2+^ sensor R-GECO1 (*Lat52_pro_::R-GECO1*) was used to measure Ca^2+^ oscillations in elongating pollen tubes. (a,b), Images of changes in cytoplasmic Ca^2+^ oscillations during growth for a WT pollen tube (a) and a *wit12* pollen tube (b). Images in a and b correspond to time points 1 and 2, shown in (c) and (d). Scale bar = 10 µm. (c), Cytoplasmic Ca^2+^ oscillations measured at the pollen tube tip of WT pollen are represented as fluorescence intensity in the blue line. The red line marks the pollen tube growth from the origin at the start of imaging. The black line represents a 4-point rolling average of the data used to highlight the overall trend. Numbers in the graph correspond to the image numbers in (a). (d), Cytoplasmic Ca^2+^ fluctuations at the tip of elongating *wit12* pollen. Fluorescence intensity is depicted by the red line. The blue line marks the pollen tube growth from the origin at the start of imaging. The black line represents a 4-point rolling average of the data used to highlight the overall trend. Numbers in the graph correspond to the image numbers in (b). (e**–**g), Cytoplasmic Ca^2+^ oscillations were quantified based on the number of peaks (e), the average fluorescence intensity for the entire time-lapse movie (f), and the standard deviation of the fluorescence intensity for the entire time-lapse movie (g). Twenty pollen tubes were analyzed in each case. Bars are standard error. N.S. indicates no significant difference by Student’s t-test.

### Pollen nuclear Ca^2+^ fluctuations correlate with the position of the nucleus

ROS-induced rupture is impaired in *wit12* but is still Ca^2+^ -dependent. Given that cytoplasmic Ca^2+^ oscillations were not altered in *wit12* but the position of the VN was, we hypothesized that a nuclear Ca^2+^ signal may play a role in ROS-induced pollen tube rupture. Increasing the distance of the VN from the pollen tube tip might thus alter a nuclear Ca^2+^ signal. Nuclear Ca^2+^ fluctuations have been described as compounds of signal transduction pathways in a variety of biological systems, notably during root symbioses [40,42]. After nodulation (Nod) or mycorrhizal (Myc) factor perception, pronounced Ca^2+^ oscillations in and around the nucleus are observed, which are required for the transcriptional response of the host plant [43]. Examples of nuclear Ca^2+^ spiking were also found in relation to biotic and abiotic stress as well as root development; however, in these cases single or few spikes were observed instead of pronounced oscillations [43,44].

**Figure 3.**
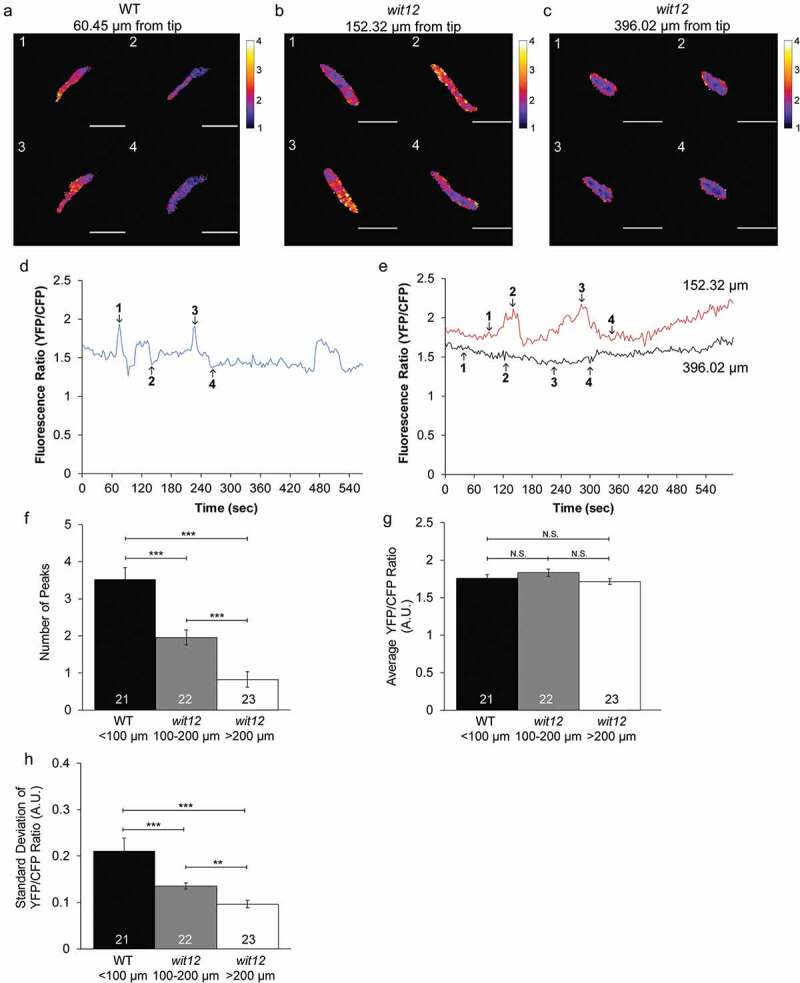
Pollen nuclear Ca^2+^ fluctuations correlate with the position of the nucleus.

To observe nuclear Ca^2+^, we generated transgenic WT and *wit12* plants expressing the VN-localized Ca^2+^ sensor NLS-YC3.6, driven by the LAT52 promoter (Figure 3a-c). Several attempts to create a *wit12* line expressing the NLS-R-GECO sensor driven by the LAT52 promoter resulted only in genetically unstable progeny. Therefore, NLS-YC3.6 was used here as nuclear Ca^2+^ sensor. We will refer to changes in nuclear Ca^2+^ as fluctuations instead of oscillations because of their irregular nature. WT nuclei exhibited clear Ca^2+^ fluctuations at 7 hpp (Figure 3d). In contrast, the Ca^2+^ fluctuations observed in elongating *wit12* pollen tubes at 7 hpp exhibited a range of patterns. In some cases, the number of Ca^2+^ fluctuations was reminiscent of those observed in WT (not shown). In other cases, the number of Ca^2+^ fluctuations was reduced compared to WT (Figure 3e, red line) or no fluctuations were observed (Figure 3e, black line).

(a–h), The nucleus-localized Ca^2+^ sensor (*Lat52_pro_*::NLS-YC3.6) was used to measure Ca^2+^ fluctuations in the VNs of elongating pollen tubes (a–c), Ratio images of changes in nuclear Ca^2+^ fluctuations during growth for a WT pollen tube with the VN positioned 60 µm from the tip (a), a *wit12* pollen tube with the VN positioned 152 µm from the tip (b), a *wit12* pollen tube with the VN positioned 396 µm from the tip (c). Scale bar = 10 µm. (d), A representative nuclear Ca^2+^ graph for a WT nucleus 60 µm from the pollen tube tip, presented as FRET ratio of YFP to CFP fluorescence intensity, is shown as a blue line. Numbers in the graph correspond to the image numbers in (a). (e), A representative nuclear Ca^2+^ graph for a *wit12* nucleus 152 µm from the pollen tube tip, presented as a FRET ratio of YFP to CFP fluorescence intensity, is shown as a red line. A representative nuclear Ca^2+^ graph for a *wit12* nucleus 396 µm from then pollen tube tip, presented as a FRET ratio of YFP to CFP fluorescence intensity, is shown as a black line. Numbers in the graph correspond to the image numbers in (b) and (c), respectively. (f–h), Nuclear Ca^2+^ fluctuations were quantified based on the number of peaks (f), the average YFP/CFP ratio for the entire time-lapse movie (g), and the standard deviation of the YFP/CFP ratio for the entire time-lapse movie (h). At least 20 pollen tubes were analyzed per background. Columns were split based on the distance of the VN from the pollen tube tip. Bars are standard error. N.S. indicates no significant difference by Student’s t-test. * P < 0.05; **P < 0.01; *** P < 0.001.

We next tested if the variation in nuclear Ca^2+^ fluctuations in *wit12* was dependent on the distance of the VN to the pollen tube tip. As in Figure 2, we used peak number and standard deviation to quantify differences in Ca^2+^ fluctuations. A Ca^2+^ peak was defined as a signal that both increased and decreased at least 0.25 A.U. For each 10-minute movie generated, the distance between the VN and tip was also recorded. WT nuclei exhibited Ca^2+^ fluctuations with an average of 3.5 peaks per 10 minutes. All VNs were positioned between 50 and 100 µm from the tip (Figure 3f-h), consistent with data presented in Figure 1j. VNs in *wit12* were distributed over a range of tip distances, but never below 100 µm. *wit12* VNs located between 100 µm and 200 µm from the pollen tube tip exhibited more WT-like Ca^2+^ fluctuations, although the average frequency of peaks per 10 minutes was reduced to 2 peaks (Figure 3f,g). *wit12* VNs located further than 200 µm from the pollen tube tip exhibited fewer Ca^2+^ fluctuations, on average showing only 1 peak (Figure 3f,g). The differences in standard deviation correspond with peak frequency (Figure 3h), with the standard deviation decreasing as the distance of the VN to the pollen tube tip increases. No significant differences in average YFP/CFP ratios between pollen tubes were observed, indicating that expression levels did not influence our results (Figure 3g). These data show that nuclear Ca^2+^ fluctuates during pollen tube growth and that the frequency of these fluctuations is correlated with the proximity of the VN to the pollen tube tip.

Finally, we examined how the cytoplasmic and nuclear Ca^2+^ levels are altered in response to H_2_O_2_. WT pollen tubes that ruptured exhibited an increase in the cytoplasmic calcium signal at the apex of the pollen tube prior to rupture (Suppl. Fig. S1, top row). This increase then propagated down the shank while the signal at the apex remained elevated (Suppl. Fig. S1 and Suppl. Movie 1). These observations are in agreement with what was described previously [26]. In *wit12* pollen tubes, a similar propagation of the Ca^2+^ signal was observed prior to burst, suggesting that there is no significant difference in the H_2_O_2_-induced tip Ca^2+^ response (Suppl. Fig. S1, bottom row and Suppl. Movie 2).

### *Nuclear Ca^2+^ fluctuations after ROS treatment are altered in* wit12

**Figure 4.**
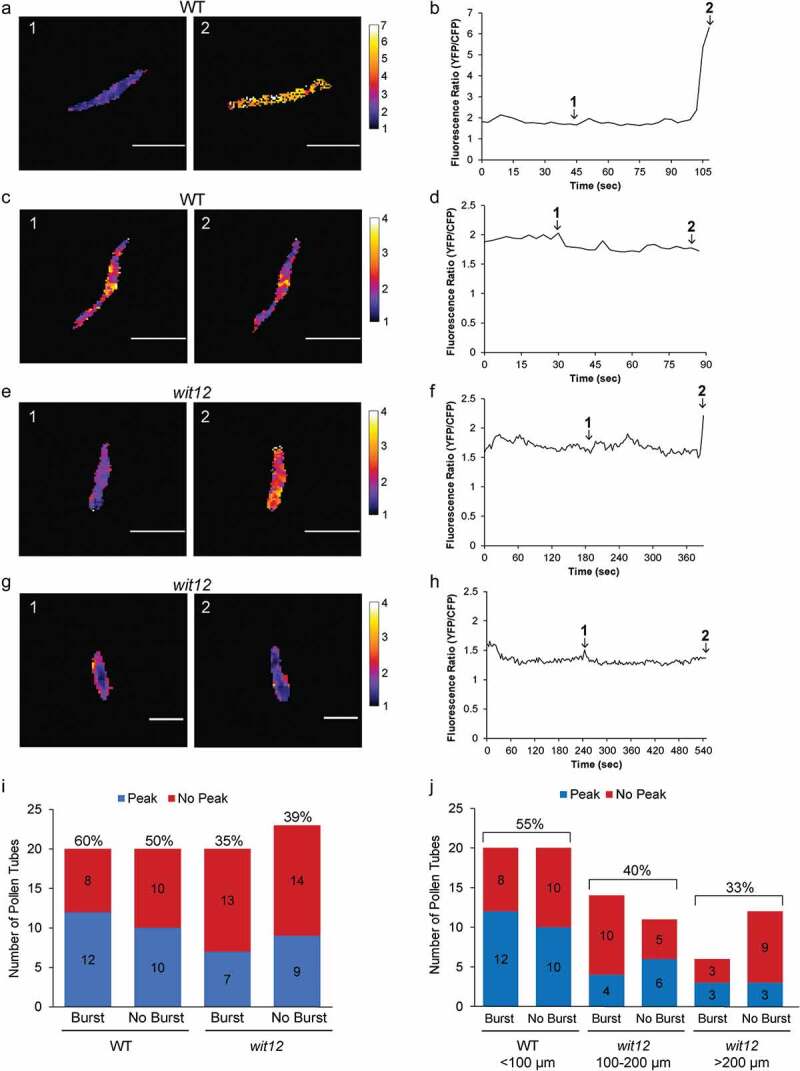
Post-ROS Ca^2+^ fluctuations.

Next, we described the nuclear Ca^2+^ fluctuations of WT and *wit12* pollen in response to H_2_O_2_. Pollen tubes, expressing the NLS-YC3.6 sensor, were treated with H_2_O_2_ at 7 hpp. A Ca^2+^ peak was defined as a signal that both increased and decreased at least 0.5 A.U., or that increased at least 0.5 A.U. immediately prior to rupture. A range of nuclear Ca^2+^ peaks were recorded, from a peak height of 7 A.U. to the lowest peak counted of 0.5 A.U. For illustration purposes, we show example ratiometric images of nuclear Ca^2+^ fluctuations prior to pollen tube rupture and corresponding graphs for WT (Figure 4a-d) and *wit12* (Figure 4e-h). The first example for both WT (Figure 4a,b) and *wit12* (Figure 4e,f) shows pollen tubes that exhibited a peak prior to rupture. The second example for both WT (Figure 4c,d) and *wit12* (Figure 4g,h) shows pollen tubes without a Ca^2+^ peak prior to rupture. Sixty percent of WT pollen tubes that burst showed a peak, compared to 50% that did show a peak but did not burst (Figure 4i). Thirty-five percent of *wit12* pollen tubes that burst and 39% of *wit12* pollen tubes that did not burst showed a peak (Figure 4i). Thus, overall fewer *wit12* pollen tubes showed a post-H_2_O_2_ nuclear Ca^2+^ peak compared to WT, but this reduction was not correlated with whether the pollen tubes burst or did not burst.

(a–j), The nucleus-localized Ca^2+^ sensor (*Lat52_pro_*::NLS-YC3.6) was used to measure Ca^2+^ fluctuations in the VNs after addition of exogenous H_2_O_2_. (a–h), Example ratiometric images of nuclear Ca^2+^ fluctuations prior to pollen tube rupture and corresponding graphs. Scale bar = 10 µm. (a,b), A WT pollen tube with a nuclear Ca^2+^ peak at 6.5 A.U. prior to rupture. (c,d), A WT pollen tube without a nuclear Ca^2+^ peak prior to rupture. (e,f), A *wit12* pollen tube with a nuclear Ca^2+^ peak at 2.5 A.U. prior to rupture. (g,h), A *wit12* pollen tube without a nuclear Ca^2+^ peak prior to rupture. Images designated as 1 and 2 in (a,c,e,g) are indicated as time points 1 and 2 in graphs (b,d,f) and (h–j), quantification of presence or absence of peaks for pollen tubes that burst and pollen tubes that fail to burst for both WT and *wit12*. The numbers of pollen tubes with or without a peak for each condition is shown in the bar graph. (i), WT is compared to the entire *wit12* population. The percent of pollen tubes that presented a nuclear Ca^2+^ peak is shown above each bar. (j), *wit12* columns were split based on the distance of the VN from the pollen tube tip. The percent of pollen tubes that presented a nuclear Ca^2+^ peak, with burst and no burst combined for each group, is shown above each bar.

Regardless of whether the pollen tube ruptured, 55% of WT pollen tubes had a Ca^2+^ peak while only 37% of *wit12* pollen tubes had a Ca^2+^ peak (Figure 4i,j). When the *wit12* population was split up based on the distance of the VN from the pollen tube tip, 40% of VNs positioned between 100 µm and 200 µm from the tip showed a Ca^2+^ peak, compared to only 33% VNs located over 200 µm from the tip (Figure 4j). In addition, of the 20 *wit12* pollen tubes that did burst, 14 had the VN between 100 µm and 200 µm and only 6 over 200 µm from the tip. Among the 23 non-bursting *wit12* pollen tubes, the VN was over 200 µm from the tip in 12 cases, again suggesting that the capacity to undergo H_2_O_2_-induced burst correlates with the position of the VN.

Together, these data suggest that there is a correlation between ROS-induced nuclear Ca^2+^ peaks and VN position, as well as between ROS-induced burst and VN position, but there is no correlation between nuclear Ca^2+^ peaks in individual pollen tubes and the capacity of the specific pollen tube to rupture.

## Discussion

The male fertility defect of mutants in *WIP* or *WIT* loci is based on reduced pollen tube burst. Duan et al. [26], have shown that exogenous H_2_O_2_ mimics the high-ROS environment of the synergids and leads to *in vitro* pollen tube burst in a calcium-dependent manner [26]. We show here that pollen tube burst based on exogenous H_2_O_2_ application is reduced to about 50% in the *wit12* mutant, consistent with the reported reduction in *in vivo* male fertility [10]. The *wit12* mutant response to H_2_O_2_ is still Ca^2+^-dependent, and the mutant shows no observable defect in pollen tube tip Ca^2+^ oscillations [39] or in the known H_2_O_2_-induced Ca^2+^ increase at the pollen tube tip [26]. Thus, the reduced response to H_2_O_2_ lies likely downstream of the known ROS-Ca^2+^ interplay at the plasma membrane that precedes pollen tube burst [45].

The reduced response to H_2_O_2_ correlates with the distance of the VN from the pollen tube tip. One hypothesis consistent with this phenomenon is that a tip-based, diffusible signal needs to be perceived by the VN for the orchestration of the pollen tube response. A relationship between position of the nucleus and the strength of a signaling pathway has been shown in animals, for example in the notch signaling pathway [46]. In this case, a diffusible compound is involved and the signal reaching the nucleus is dampened by increased distance. Reception of the signal in this system also requires a LINC complex for nuclear movement [10,46]. In Arabidopsis pollen, mutants in a family of transcription factors (MYB97/101/120) show similar pollen tube reception defects as those observed in *wit12* and *wifi* mutants [17,47]. A diffusible signal might thus be upstream of the VN gene expression profile required for late stages of pollen tube growth.

Ca^2+^ as a diffusible second messenger is well established in both animal and plant systems [48,49] and was thus tested here as a possible signal. We found that neither the previously described Ca^2+^ fluctuations during pollen tube growth [39] nor the H_2_O_2_-induced rapid and expanding increase of tip-based Ca^2+^ [26] were measurably affected in the *wit12* mutant. While nuclear Ca^2+^ oscillations have now been described in a variety of biological systems [43], nuclear Ca^2+^ has, to our knowledge, not been addressed in the pollen tube nucleus. Using the ratiometric fluorescent Ca^2+^ sensor YC3.6 fused to an NLS, we describe here distinct nuclear Ca^2+^ fluctuations both during pollen tube growth and during response to H_2_O_2._ In both cases, the nuclear Ca^2+^ fluctuations were dampened in the *wit12* mutant and were further reduced in those pollen tubes with a nucleus positioned further away from the pollen tube tip. This suggests that the proximity of the VN to the pollen tube tip, and thus – by extension – to the cytoplasmic Ca^2+^ signal, is required for the establishment of the nuclear Ca^2+^ patterns.

Dual, rhythmic Ca^2+^ oscillations, at both the nuclear and cytoplasmic surface of the nuclear envelope, are a hallmark during root symbioses, and their coordination and crosstalk are an area of active investigation [40,42]. However, a number of other stimuli lead to single or few, less rhythmic calcium peaks, similar to the ones observed here [43,44]. For example, using dual cytoplasmic and nucleoplasmic GECO-based calcium sensors, Kelner et al. [50] found after cold shock and NaCl treatment in Arabidopsis root epidermal cells both a cytoplasmic and a nucleoplasmic transient calcium increase, which could be distinguished by the specific time delays between the cytoplasmic and nuclear peaks [50]. This is consistent with a study by Huang et al. [51], who showed that cytoplasmic and nuclear calcium responses to osmotic and salt stress can occur independent from each other [51]. Several earlier studies also suggest that plant nuclei can generate Ca^2+^ oscillations independent of changes in cytoplasmic Ca^2+^, and that an increase of the extranuclear Ca^2+^ concentration is not sufficient to trigger Ca^2+^ increases in isolated nuclei [52,53].

Together, this makes it unlikely that the nuclear Ca^2+^ increases observed here in the growing pollen tube ‘merely’ reflect the known cytoplasmic Ca^2+^ fluctuations by passive Ca^2+^ influx, especially in light of the fact that the frequency of fluctuation differs markedly between nucleus and cytoplasm (compare Figures 2 and 3). They are thus more likely indicative of a separate Ca^2+^ release from nuclear storage, e.g. the nuclear envelope lumen. Nevertheless, a cytoplasmic Ca^2+^ signal could be required to directly or indirectly activate a nuclear Ca^2+^ signal [43], and the distance of the nucleus from the tip could, therefore, dampen this response. Newly developed dual Ca^2+^ sensors [50] will now be better equipped to resolve the timing relationship between cytoplasmic and nuclear Ca^2+^ signatures and the relationship between the spread of cytoplasmic Ca^2+^ in the pollen tube shank and the position of the nucleus. In addition, specific nuclear and cytoplasmic Ca^2+^ quenching, as demonstrated by Huang et al. [51], can now be used to address the interdependence of the two signals.

The relationship at the single-cell level between the VN Ca^2+^ peaks and H_2_O_2_-induced pollen tube burst remains currently unclear. It is possible that the VN senses cytoplasmic Ca^2+^ signatures, including those elevated by H_2_O_2_, and that the nuclear Ca^2+^ peaks trigger unknown downstream signals, but that these are unrelated to pollen tube burst. Discovering and investigating male gametophyte-expressed nuclear envelope-associated Ca^2+^ channels will therefore now be crucial to address if the VN Ca^2+^ fluctuations are caused by nuclear envelope Ca^2+^ release and – if so – which pollen tube processes are affected by their disruption.

The movement of the vegetative nucleus through the pollen tube is one of the longest nuclear migrations in plants [54]. Nuclei move over significant distances also in other elongated plant cells, for example in root hairs and root epidermal cells. Notably, plant nuclei in these cell types are hyper-elongated and it has been proposed that this shape might protect the nuclei from physical strain during migration [54]. The elongated shape requires the WIT-WIP-SUN LINC complex, plant lamin-like CRWN proteins [19] and other nuclear envelope-associated proteins [55]. In the respective mutants, both nuclear shape and nuclear movement are affected, and this correlation is so strong that nuclear shape change has been used as a primary genetic screen to identify nuclear movement mutants [9]. Building on prior work [10], we show here that loss of the WIT-WIP-SUN LINC complex leads to a defect in nuclear migration in pollen tubes (see Figure 1). In addition, we noted that *wit12* nuclei appeared less elongated than WT nuclei, which were thin and highly stretched (see Figures 1f-i, 3a-c, 4a-g, S2A). When quantified by calculating the circularity index for WT and *wit12* vegetative nuclei, it became apparent that *wit12* nuclei are indeed shorter and more circular than WT nuclei, consistent with similar nuclear shape changes observed in the *wit12* sporophyte (Suppl. Fig. S2B). This reduced elongation might be a consequence of reduced forces acting on the *wit12* VN, which might be released from an active transport process in the pollen tube.

A number of older studies in species other than Arabidopsis suggest that cytoskeletal motors might be involved in pollen VN transport. *Galanthus nivalis* pollen tubes show the VN preceding the SC, similar to Arabidopsis. Depolymerization of microtubules affects this order and leads to an increased distance between the VN and the pollen tube tip [56]. In addition, depolymerizing *Lilium henryi* F-actin leads to a contraction of the elongated VN, and a greater distance between the VN and SCs [57]. Inhibitor experiments like the ones described in these studies are difficult to use in a way that allows to clearly separate effects on pollen tube elongation and VN movement. Instead, a search for Arabidopsis pollen-expressed motor proteins that bind to the WIT-WIP-SUN LINC complex and have an effect on VN shape, VN movement and male fertility would be fruitful to dissect the important mechanism of keeping the VN close to the pollen tube tip during all stages of pollen tube development. It is formally possible that the dampened ROS and Ca^2+^ responses in *wit12* are not a result of increased distance between VN and pollen tube tip, but stem from an unknown role of the WIT-WIP-SUN LINC complex other that nuclear shape and movement control. Identifying motor mutants that recapitulate the ROS and Ca^2+^-related effects described here could therefore functionally tie the role of the LINC complex in nuclear movement to its roles in ROS response and nuclear Ca^2+^ response.

## Supplementary Material

Supplemental Material

## Accession numbers

Sequence data from this article can be found in the GenBank/EMBL data libraries under accession numbers NM_001160797 (WIP1), NM_125004 (WIP2), NM_112181 (WIP3), NM_121177 (WIT1), and NM_105565 (WIT2).
