# Supplementary material for: LINC-complex mediated positioning of the vegetative nucleus is involved in calcium and ROS signaling in Arabidopsis pollen tubes
Source: Nucleus. 2020 Jul 7;11(1):149–63. doi: 10.1080/19491034.2020.1783783 (PMC7529407; doi:10.1080/19491034.2020.1783783)
Supplement: Supplemental Material [file KNCL_A_1783783_SM1057.zip › Supplementary information/Supplemental legends.docx]

**Supplemental Data**

The following materials are available in the online version of this article.

**Supplemental Figure S1: ROS-induced cytoplasmic Ca^2+^ oscillations are not disrupted in *wit12* pollen tubes.** The Ca^2+^ sensor R-GECO1 (*Lat52_pro_::R-GECO1*) was used to measure cytoplasmic Ca^2+^ oscillations over time in pollen tubes after addition of H_2_O_2_. Representative images of changes in cytoplasmic Ca^2+^ fluctuations for one WT pollen tube (top panel) and one *wit12* pollen tube (bottom panel). The white dotted line outlines the pollen tube wall using the corresponding DIC image. Numbers in the bottom left corner are the RFP fluorescence of the corresponding ROIs (yellow circles), located close to the pollen tube tip. Time 0 indicates the start of imaging after H_2_O_2_ addition. Imaging sequences shown are representative of several videos for each genotype. Scale bar = 10 µm.

**Supplemental Figure S2: *wit12* nuclei are more circular than WT nuclei. A**, Representative images of a WT VN (top) and a *wit12* VN (bottom) at 7 hpp, using the YFP signal of NLS-YC3.6 as a nuclear marker. **B**, Circularity index of vegetative nuclei from WT and *wit12* pollen tubes. (Left) Scatter plot showing each nucleus as a single point. (Right) Box plot with Top line, Maximum; Box, Quartiles; Solid middle line, Median; Dotted middle line, Mean; and Bottom line, Bottom fence. ***P<0.001 for Student’s t-test.

**Supplemental Table S1*:* Primers used for cloning*.*** CACC sites for directional TOPO cloning are indicated in bold. Specific recognition sites for *SacI* and *SpeI* are underlined.

**Supplemental Movie S1**

An example of cytoplasmic Ca^2+^ oscillations in a WT pollen tube after addition of H_2_O_2_. The pollen tube of interest is to the right of the white circle. The Ca^2+^ sensor R-GECO1 (*Lat52_pro_::R-GECO1)* was used to measure changes in Ca^2+^ over time. The pollen tube tip was imaged every 5 seconds until rupture. Movie playback speed is 60x.

**Supplemental Movie S2**

An example of cytoplasmic Ca^2+^ oscillations in a *wit12* pollen tube after addition of H_2_O_2_. The pollen tube of interest is to the right of the white circle. The Ca^2+^ sensor R-GECO1 (*Lat52_pro_::R-GECO1)* was used to measure changes in Ca^2+^ over time. The pollen tube tip was imaged every 5 seconds until rupture. Movie playback speed is 60x.
