# Supplementary material for: LINC-complex mediated positioning of the vegetative nucleus is involved in calcium and ROS signaling in Arabidopsis pollen tubes
Source: Nucleus. 2020 Jul 7;11(1):149–63. doi: 10.1080/19491034.2020.1783783 (PMC7529407; doi:10.1080/19491034.2020.1783783)
Supplement: Supplemental Material [file KNCL_A_1783783_SM1057.zip › Supplementary information/Supplemental Table 1.pdf]

Supplemental Table 1: Primers used for cloning

Primer Name	Sequence (5' – 3')	Construct
Lat52ProSacIF	aaagagctccctataccccttggataaggg	Lat52 promoter
Lat52ProSpeIR	ttactagttttaaattggaattttttttttttggtg	Lat52 promoter
NLS-YC3.6F	**cacc**atgctgcagcctaagaagaa	NLS-YC3.6 calcium sensor
NLS-YC3.6R	ggggaccactttgtacaagaaagctgggtattactcgatgttgtggcgg	NLS-YC3.6 calcium sensor
R-GECO1F	**cacc**atggtcgactcttcacgtcgtaa	R-GECO1 calcium sensor
R-GECO1R	ctacttcgctgtcatcatttgtaca	R-GECO1 calcium sensor

CACC for directional Topo cloning are bold
Specific recongition sites for *SacI* and *SpeI* are underlined
