# Supplementary figures and images for: LINC-complex mediated positioning of the vegetative nucleus is involved in calcium and ROS signaling in Arabidopsis pollen tubes
Source: Nucleus. 2020 Jul 7;11(1):149–63. doi: 10.1080/19491034.2020.1783783 (PMC7529407; doi:10.1080/19491034.2020.1783783)

Supplemental Figure 1

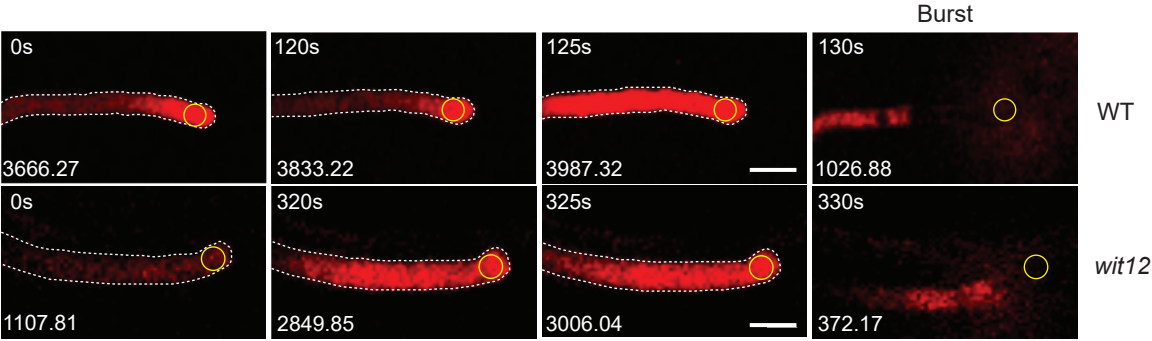

Supplement: Supplemental Material [file KNCL_A_1783783_SM1057.zip › Supplementary information/Supplemental Figure 1 (1).pdf]

Supplemental Figure 2

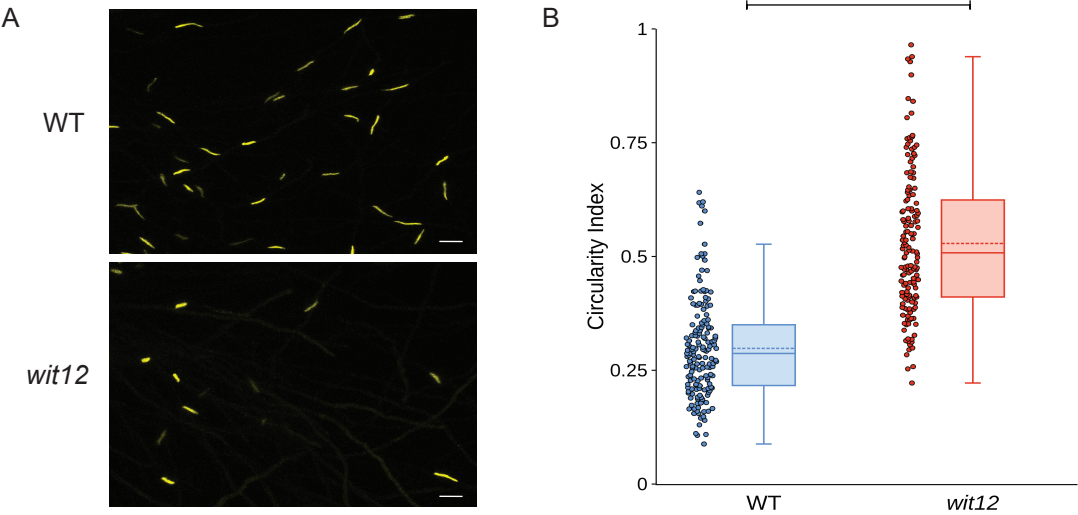

Supplement: Supplemental Material [file KNCL_A_1783783_SM1057.zip › Supplementary information/Supplemental Figure 2 (1).pdf]
